# Are women better mindreaders? Sex differences in neural correlates of mentalizing detected with functional MRI

**DOI:** 10.1186/1471-2202-10-9

**Published:** 2009-02-04

**Authors:** Sören Krach, Isabelle Blümel, Dominic Marjoram, Tineke Lataster, Lydia Krabbendam, Jochen Weber, Jim van Os, Tilo Kircher

**Affiliations:** 1 Department of Psychiatry und Psychotherapy, Section of Neuroimaging, Philipps-University Marburg, Rudolf-Bultmann-Straße 8, D-35039 Marburg, Germany; 2 Department of Psychiatry und Psychotherapy, Philipps-University Marburg, Rudolf-Bultmann-Straße 8, D-35039 Marburg, Germany; 3 Department of Psychiatry, RWTH Aachen University Hospital, Pauwelsstr. 30, D-52074 Aachen, Germany; 4 Department of Psychology, University of Glasgow, 58 Hillhead Street, Glasgow, G12 8QB, UK; 5 Department of Psychiatry and Neuropsychology, Maastricht University, PO BOX 616 (location DOT10) 6200 MD Maastricht, the Netherlands; 6 Social Cognitive Neuroscience Laboratory Department of Psychology, Columbia University, 1190 Amsterdam Avenue, New York City, NY 10027, USA

## Abstract

**Background:**

The ability to mentalize, i.e. develop a Theory of Mind (ToM), enables us to anticipate and build a model of the thoughts, emotions and intentions of others. It has long been hypothesised that women differ from men in their mentalizing abilities. In the present fMRI study we examined the impact of (1) gender (women vs. men) and (2) game partner (human vs. computer) on ToM associated neural activity in the medial prefrontal cortex. Groups of men (n = 12) and women (n = 12) interacted in an iterated classical prisoner's dilemma forced choice situation with alleged human and computer partners who were outside the scanner.

**Results:**

Both the conditions of playing against putative human as well as computer partners led to activity increases in mPFC, ACC and rTPJ, constituting the classic ToM network. However, mPFC/ACC activity was more pronounced when participants believed they were playing against the alleged human partner. Differences in the medial frontal lobe activation related to the sex of the participants could be demonstrated for the human partner > computer partner contrast.

**Conclusion:**

Our data demonstrate differences in medial prefrontal brain activation during a ToM task depending on both the gender of participants and the game partner.

## Background

Folk psychological opinion views women as being more sensitive, emotional and even better mind readers as compared to men. In a scientific context these abilities refer to empathy and the adoption of a Theory-of-Mind (ToM). Empathy describes the sensitive perception of feelings and emotions of others [1], while Theory-of-Mind refers to the more cognitive aspect of inferring intentions, goals or desires of others [2]. From an evolutionary perspective, having a ToM provides a powerful selective advantage, since it means the intentions of a counterpart can be evaluated in advance and responded to adequately. In highly developed societies appropriate social interactive behaviour in everyday situations (i.e. working environment, private and family life etc.) is regarded as a key cognitive ability and women are often regarded as superior in this respect.

In recent years, functional neuroimaging studies have increasingly addressed Theory-of-Mind related questions, including irony [3], jokes [4,5], envy [6], false beliefs [7] and embarrassment [8]. Other approaches utilize games and simulations that enable implicit and online ToM mechanisms to be investigated [9-16]. Here, the experimenter takes advantage of the distraction of the proper game, which puts the participants into a "flow-like" state and implicitly prevents the participant to direct their attention to the actual question of the taking of the perspective of a respective game partner on the basis of "I think that you think that I think... etc." Task designs applied to study implicit mentalizing comprise the Prisoner's Dilemma Game (PDG) [9,11,16,17], the Ultimatum Game [11], economic decision games (Iowa Gambling Task) [15,18] or a version of the stone-paper-scissor game [14].

The neural substrates associated with mentalizing comprise circumscribed regions of the right temporo-parietal junction (TPJ) and the medial prefrontal gyrus extending into the anterior (para)-cingulate cortex (ACC) [19-24]. The TPJ has been shown to be activated in relation to biological motion and to stimuli which signal intentions and intentional activity [22-25]. On the other hand, medial prefrontal and anterior cingulate regions are associated with planning and anticipation [26]. Frith and Frith assume that in the context of mentalizing these regions play a major role in the anticipation of what and of how a partner is feeling, thinking or doing on the basis of what oneself would feel, think or do in the same situation [19,27]. In turn, this shift permits the assessing and evaluating of ones own feelings and thoughts on a highly self-reflective level [28,29].

Recent studies have shown that we also implicitly attribute intentions of various degrees to animals [30,31] and to a somewhat lesser extent even to robots [9,32] and computers [11]. Neuroimaging findings indicate that humans do attribute self-generated actions, intentions and desires more greatly to human than to computer partners, though activity in the mentalizing network was detected in human-computer interactions as well. This was most evident in scenarios when the computer/machine was perceived as being directly responsive to the subjects' behavioural decisions [9,11,14,17,32].

The influence of gender on ToM along with its neural correlates has hardly been investigated. Accordingly, debates on this issue have been dominated by folk psychological positions and merely favour the point of view that women are superior in mentalizing and associated abilities.

In the present study we focused on the questions of whether humans attribute intentions and goals to human vs. computer partners and whether the participants' gender modifies neural correlates of implicit mentalizing. We applied a version of the PDG with subjects instructed to play either a putative human or a computer partner (while actually both were programmed to "play" a random sequence). By means of a cover story we were able to infer pure "intentional stance" [14,33] associated neural activity as possible strategies of both partners were held constant between conditions. We hypothesized that subjects would attribute more real intentions and thoughts to the human partner, while the assumed random answer of the computer would elicit less brain activity in the medial prefrontal cortex. Regarding sex differences, we expected, in line with folk psychological assumptions that women would be better perspective takers and therefore display stronger signal changes in the medial prefrontal cortex.

## Results

### Behavioral results

Reaction times and averaged accumulated pay-off differences are listed in Table 1. The reaction time data were entered into repeated measures ANOVA with sex as between-subject factor and game partner as within-subject factor. Results revealed that there was no effect of sex (*F *(1, 21) = 0.36, *p *= .56) and partner (*F *(1, 21) = 3.56, *p *= .07) and no interaction between sex and game partner (*F *(1, 21) = 0.52, *p *= .48).

**Table 1:** Behavioural Data

	**♂ = 12**		**♀ = 12**	
	**M**	**SD**	**M**	**SD**
Age	28.0	5.5	26.1	6.0
IQ	114.4	14.3	119.3	14.3
RT (playing against computer partner) (ms)	388.2	101.7	417.7	107.7
RT (playing against human partner) (ms)	405.2	101.7	425.3	91.6
Pay-off computer (playing against computer partner) [points]	360.0	172.4	298.2	107.3
Pay-off subject (playing against computer partner) [points]	803.3	72.4	769.1	104.6
Pay-off computer (playing against human partner) [points]	476.7	133.5	328.2	127.6
Pay-off subject (playing against human partner) [points]	775.0	111.3	808.2	83.4
Questionnaire: Did you have the impression to play against another person? (no, not at all = 1; yes, very much = 7)	5.0	1.7	5.2	1.2

Accumulated pay-off's during the participant-computer as well as participants-human partner interactions were then entered into a repeated measures ANOVA with sex as between-subject factor and game partner as within-subject factor. Results revealed no effect of sex (*F *(1, 21) = 0.00, *p *= .97) and partner (*F *(1, 21) = 0.43, *p *= .83) and no interaction between sex and game partner (*F *(1, 21) = 1.69, *p *= .21).

When the points given to the respective partner (either putative human partner or computer partner) by players were investigated, a significant sex difference emerged in the respect that females played more competitively, indicated by increased pressing of the right response button [repeated measures ANOVA: effect of sex (*F *(1, 21) = 6.60, *p *= .02) and partner (*F *(1, 21) = 3.26, *p *= .09) and no interaction between sex and game partner (*F *(1, 21) = 1.14, *p *= .30)].

Overall, both male and female subjects gained significantly more points than the alleged game partner, using a very competitive strategy on average [repeated measures ANOVA human condition: effect of sex (*F *(1, 21) = 3.30, *p *= .08) and partner (*F *(1, 21) = 113.31, *p *= .00) and no interaction between sex and game partner (*F *(1, 21) = 6.17, *p *= .02); repeated measures ANOVA computer condition: effect of sex (*F *(1, 21) = 3.14, *p *= .09) and partner (*F *(1, 21) = 116.57, *p *= .00) and no interaction between sex and game partner (*F *(1, 21) = 0.10, *p *= .75)].

The questionnaire handed out after scanning revealed that all subjects regardless of sex had been completely convinced that they were playing a real human contender in the "human condition" and therefore validated the successful "deception" (see Table 1). Only two subjects admitted seeing through the cover story and were therefore discarded from later fMRI data analyses.

### Neuroimaging results

#### Effects of game partner

Second-level group effects (human/computer partner > baseline) brain activity differed with respect to the partner being played. Activity modulation during the contrast "human partner > baseline" comprised a wide-spread network of middle frontal, superior medial frontal and inferior parietal regions (see table 2). Areas involved during "computer partner > baseline" centred on the middle frontal cortex extending into the inferior parietal cortex (see table 2). The direct contrast of both experimental conditions revealed circumscribed activations of medial frontal areas only for "human partner > computer partner". The reversed contrast "computer partner > human partner" did not yield any significant ToM related activity. It is important to note that all inferior parietal cortex activations as well as lateralized frontal activity documented above by applying simple contrasts (see Table 2) were subtracted out in this complex contrast.

**Table 2:** Significance level and the size of the respective activation cluster (number of voxels) for male and female (human partner > baseline; computer partner > baseline).

		**Coordinates**				
	**BA**	**x**	**Y**	**z**	**t-value**	**No. voxels**
**Male (human partner > baseline)**						

L Inferior Frontal Gyrus (orbital part) L Insula	8/10	32	19	-8	10.94	47
L Middle Frontal Gyrus	8	51	17	29	10.48	63
L Middle Frontal Gyrus	8	36	47	9	9.42	46
L Superior Frontal Gyrus (medial part)	9	8	29	35	9.20	28
L Inferior Parietal Lobule	7/40	55	-44	46	8.68	93
R Angular Gyrus Gyrus R Superior Parietal Lobule	39/40	-28	-56	47	7.80	10
R Inferior Parietal Lobule	39/40	-40	-41	35	7.75	19
L Middle Frontal Gyrus L Precentral	8	40	18	54	7.44	27

**Male (computer partner > baseline)**						

L Middle Frontal Gyrus	8	36	55	5	10.72	110
L Superior Frontal Gyrus (medial part) L Superior Frontal Gyrus	9	8	29	35	10.50	24
R Angular Gyrus R Inferior Parietal Lobule	39/40	-44	-41	39	9.71	45
L Inferior Frontal Gyrus (orbital part) L Insula L Superior Temporal Pole	11/44/45	40	23	-15	9.67	26
L Inferior Parietal Lobule	39/40	40	-56	47	8.25	122
R Middle Frontal Gyrus (orbital part)	8/10	-40	50	-13	7.96	78
R Middle Frontal Gyrus	8	-40	54	-3	7.89	10
L Superior Parietal Lobule	7	28	-71	55	7.53	30

**Female (human partner > baseline)**						

L Inferior Parietal Lobule	39/40	48	-45	28	8.87	83
L Supplementary Motor Area L Superior Frontal Gyrus (medial part)	6/10	0	25	39	8.17	14

**Female (computer partner > baseline)**						

L Middle Frontal Gyrus	8	48	37	31	10.22	23
L Inferior Parietal Gyrus	39/40	51	-44	43	8.94	97

Only clusters of at least 10 voxels are shown (uncorrected for multiple comparisons at p < .0001).

#### Effects of gender

**Figure 1:**
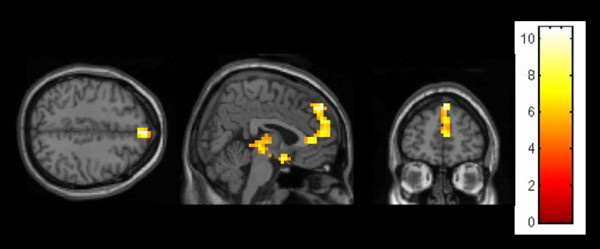
**Procedure of the fMRI setting**. Stimuli display and time course of the applied paradigm.

**Figure 2:**
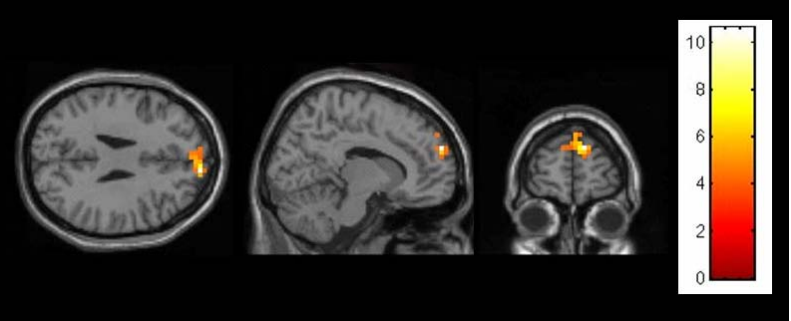
**Male (human partner > computer partner) showing a peak activation in the medial prefrontal gyrus (FWE p < .05, Monte Carlo corrected)**.

These findings proved to be independent of the subjects' gender (see Table 3; Figures 1 and 2). However, results indicate a significantly pronounced engagement of medial frontal regions as well as the thalamic region in the male relative to the female cohort. Furthermore, the local maxima activation within the medial frontal cortex was located somewhat superior in males relative to females (male: z = 38; female z = 22).

**Table 3:** Significance level and the size of the respective ToM relevant activation cluster (number of voxels) for male and female (person > computer) and male > female (person > computer).

		**Coordinates**				
				
	**BA**	**x**	**Y**	**z**	**t-value**	**No. voxels**
**Male (person > computer)**						
R Superior Frontal Gyrus (medial part) R Anterior Cingulate Cortex	9/32	4	52	38	9.13	113
		4	35	2	7.39	
		4	55	12	6.97	
L/R Thalamus		12	-27	1	7.95	96
		4	-23	1	6.15	
		-8	-20	-6	5.94	
R Olfactory Cortex	25	4	3	-14	7.41	18
L Cerebellar Cortex		-28	-75	-20	6.34	42
		-24	-87	-23	6.03	
		-36	-79	-23	5.59	
L Cerebellar Cortex		-8	-52	-31	5.96	9
R Middle Temporal Gyrus R Inferior Temporal Gyrus	21	59	-16	-16	5.92	8
R Cerebellar Cortex		44	-71	-17	5.21	6
R Superior Frontal Gyrus R Supplementary Motor Area	8	4	30	50	5.17	6
R Anterior Cingulate Cortex	32	0	32	21	5.13	7
**Female (person > computer)**						

L/R Superior Frontal Gyrus (medial part)	10/9	12	59	23	10.58	40
		16	56	34	5.77	
		-4	51	20	5.57	
R Middle Temporal Gyrus R Angular Gyrus	40/39	59	-53	21	8.43	21
		51	-61	25	4.85	
L Cerebellar Cortex		-32	-79	-30	6.86	10
R Posterior Cingulate Cortex	31	-8	-45	35	5.91	15
		0	-49	36	5.61	
		-8	-44	43	4.53	

**Male > female (person > computer)**						

R Anterior Cingulate Cortex L Superior Frontal Gyrus (medial part)	32	8	39	5	5.30	11
L Cerebellar Cortex		-28	-71	-20	4.40	7

Only clusters of at least 6 voxels are shown (FWE p < .05, Monte Carlo corrected for multiple comparisons at p < .0001).

#### Interaction of gender and game partner

**Figure 3:**
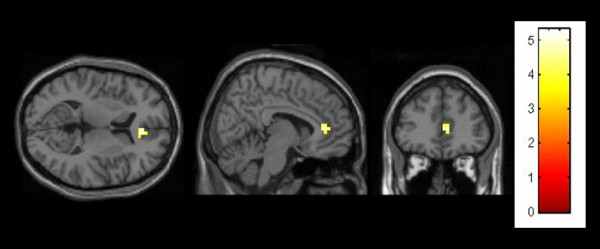
**Female (human partner > computer partner) showing a peak activation in the medial prefrontal gyrus (FWE p < .05, Monte Carlo corrected)**.

We further directly investigated sex differences for the complex contrasts. Under the contrast "human partner > computer partner" only two activation clusters reached significance when activity modulation in males was contrasted with females [male > female (human partner > computer partner)]: the right anterior cingulate gyrus extending into the medial frontal cortex as well as a small region in the left cerebellar cortex (see Figure 3). Parameter estimates extracted at the local maximum activation in the ACC region [x = 8, y = 39, z = 5] neither correlated with pay-off outcomes during the human-computer nor during the human-human interaction (r_human-computer _= .07; p > .05; r_human-human _= .26; p > .05).

In addition, by reversely contrasting female with male subjects (under the same presumption) no region was activated significantly.

## Discussion

With the present fMRI study we aimed at elucidating the impact of (1) gender (women vs. men) and (2) interaction partner (human vs. computer) on brain activation in the medial frontal lobe as a correlate of Theory-of-Mind processes. Groups of male and female subjects played an iterated version of the classical Prisoner's Dilemma Game (PDG) against either a putative human or computer partner. A cover story helped to mislead subjects to assume that they were playing a real human partner, though both games were "played" by a pre-programmed randomized decision sequence. The success of the deceit could be verified by a post-hoc questionnaire. As possible confounds by differing strategic behaviour of a real partner were thus held constant, neural activity provided a direct measure of pure intentional stance [14,33]. In summary, we could demonstrate differences in medial prefrontal brain activation during a ToM task, depending on the gender of participants and the alleged game partner.

Regarding the results with respect to the partner being played, our data indicate that regions previously associated with ToM tasks (i.e. mPFC extending into the ACC) have been evoked more strongly by human contenders as opposed to computer partners [9,20,21]. This finding is independent of the decision process as the reaction times were equal across conditions. Thus, our results confirm findings of Rilling and colleagues who likewise reported differential brain activations with respect to the interaction partner (human or computer) being played [11]. Our main finding of ACC/superior medial frontal gyrus activation in both cohorts (for the complex contrast human partner > computer partner) is also in fitting with the findings of Gallagher and colleagues (2002). In their study – subjects played a computerized version of the children's "stone-paper-scissor" game against either human or computer partners – they detected highly overlapping activation centres (coordinates of anterior paracingulate cortex in Gallagher et al. (2002): x = 8/-10; y = 54/50; z = 12/30; coordinates of superior medial frontal activation in our study (male/female): x = 4/12; y = 52/59; z = 38/23) when computing comparable contrasts. Overall, the anterior cingulate/paracingulate cortex (ACC), a region rather difficult to dissociate from the medial prefrontal cortex, has been consistently associated with tasks necessitating mentalizing performance [9,11,12,15,16,18-21,34]. Other neuroimaging studies link ACC/medial prefrontal cortex functioning to uncertainty arousal [35,36], cognitive conflict [37], future planning and anticipation [38], self-monitoring [39] or self-recognition [40]. Common to all of the applied paradigms is that they require the reflection of mental states of oneself and others, as mentioned above, an ability preferably ascribed to women as opposed to men. We demonstrated differential activation in the medial prefrontal cortex for the human > computer partner condition between groups. If we assume that this region is correlated with ToM processes, we may conclude gender specific activation. The following reasons may contribute to this difference: (1) women were not as engaged playing an alleged soulless computer, however the behavioural data imply against this assumption; (2) men were compensating for weaker ToM abilities by increased effort, resulting in the stronger activation. Again, this can not be confirmed by the behavioural results; and (3) male and female subjects always played a male contender in the "human partner" condition. As documented previously, in the presence of a male partner men and women play games differently [41-43], suspecting considerably varying activity patterns depending on the gender of confederates as well as subjects. In order to rule out such effects we correlated cortical activity measures (parameter estimates derived at the local maxima activation of the contrast male > female [human partner > computer partner]) with behavioural measures (pay-off outcomes) and could not find any significant effect. Thus, neural correlates of mental state attribution may not be explained by an interaction with behavioural measures, but rather display fundamental differences in utilizing ToM relevant structures in a direct interaction with same-sex/different-sex game partners. Admittedly, to directly address this question one may have needed to engage both gender groups with a female as well as a male confederate.

Finally, additional signal changes in the right temporo-parietal junction (TPJ), as various studies on related ToM tasks have documented [22-24], might have been expected. TPJ activity, however, obvious in the baseline contrast, was similar during games with the human and the computer partner (with right hemisphere > left hemisphere). Hence, TPJ related activity was subtracted out in the direct comparison of human > computer partner. We therefore argue that the TPJ activity is only secondary with respect to the game partner being played and rather displays a somewhat general attribution of behaviour to another agent (and the analysis of the goals and outcomes of such behaviours).

In conclusion, we engaged groups of men and women in a real life reciprocal and iterated interaction task with another human and a computer. We detected stronger mPFC activity for human-human as opposed to the human-computer interactions, which was independent of the subjects' gender. More interestingly, we demonstrate differences in medial prefrontal brain activation during the ToM task, depending on the gender of participants and the alleged game partner. The exact reason for this activation difference and its consequence requires further elucidation in further studies.

## Methods

### Subjects

24 (12 female, 12 male) native German subjects of Western- or Middle European descent participated in the study. The mean age of the participants was 27.4 years, with a range from 19 to 40 years. In order to control for possible cognitive factors that may influence the performance on the task, neuropsychological testing comprising executive functions [44] and IQ [45] was administered (see table 1). Age, executive functions and IQ did not differ significantly between sexes. All participants were students or employees recruited from the University Hospital Aachen. The educational status of men and women was matched in years spent at school and university. All subjects had normal or corrected-to-normal vision and were right-handed according to the Edinburgh Handedness Index [46]. Subjects were excluded if they were diagnosed according to ICD-10 with a past or present psychiatric, neurological, or medical disease as well as with psycho-pharmacological medication intake at time of study or within the previous two months. The study was approved by the local ethics committee according to the declaration of Helsinki. All participants signed written informed consent prior to participation and were paid a fee for participation.

### Stimuli and task design

Prior to scanning, all subjects completed a briefing consisting of three tutorial rounds for each condition in order to familiarize subjects with the decision matrix. The decision matrix resembled matrices already applied by other research groups and is considered as a variant of the PDG [11,12,16]. In short, subjects were informed that if both contenders (subject vs. human partner or subject vs. computer partner) were pressing the left button, both of them would be gratified with 10 points each (CC). In the case that the subject would press the left button (cooperate) with the partner pressing the right button at the same time (defect), the subject would return empty-handed for this game while the partner would receive 20 points (CD). Conversely, the subject could defect and would reap 20 points, whilst the non-defecting partner would get zero points (DC). In the case that both contenders chose to defect, the dilemma would eventuate with both sides receiving zero points (DD). CC implies mutual cooperation, while DD involves mutual non-cooperation [11].

The setting of the briefing was as follows: each subject was seated face-to-face with a confederate (always the same male person) with both having a commercial notebook laptop located at their side of the table. Both notebooks were linked by a connecting cable. The experimenter introduced subject and confederate and explained the task design. The first condition comprised a series of nine single games (equalling one round) with the subject playing against the confederate (*human partner*). For each single game the subject had to make a decision about cooperating or defecting with the partner. Cooperation was signalled by pressing the left button (←) on the computer keyboard, defection by pressing the right button (→), respectively. For the second condition subjects were instructed to play against the computer, again consisting of nine single games (*computer partner*). The possible response selection equalled the human partner condition. During the tutorial both conditions were presented twice in random order, interspersed by a low level "baseline condition" that enforced subjects to alternately press the right and left button when a central cross appeared on the computer screen. Furthermore, subjects were confronted with two converse goals: on the one hand subjects where enforced to win the series, while on the other hand subjects had to reach a virtual highscore. In a pre-testing scenario involving four different winning matrices the selected scenario proved to be the best to enforce subjects to vary their responses with respect to their accumulated pay-off.

**Figure 4:**
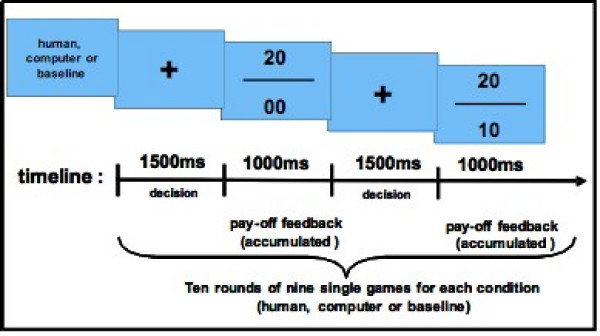
**Male > female (human partner > computer partner) revealing only one ToM related brain region to be activated differentially between sexes: the medial prefrontal cortex (FWE p < .05, Monte Carlo corrected)**.

At the beginning of each series subjects were informed about the condition to be followed (human, computer or baseline) via the computer screen. Immediately after this instruction, a central cross on the computer screen indicated the start of a nine game series and enforced the subjects to make their decision (left or right button press; see above). The central cross disappeared after 1500 ms and was followed by an accumulated pay-off feedback for the current series (1000 ms) (see Fig. 4). The accumulated pay-off feedback enabled subjects to draw exact inferences about the response selection of the partner (i.e. human or computer). The subjects' pay-off was indicated by the lower number, the partners' pay-off by the upper number, respectively. During the low level baseline no numeral response feedback was given, instead two crosses replaced the numbers on the upper and lower side of the bar.

Unknowingly, the subjects always played against a random sequence "partner", never giving the subjects an opportunity to really cooperate or find "a best way". This misleading enabled the hemodynamic changes related to differences in the instruction (human or computer partner) only to be calculated, ruling out possible interaction effects of scattered strategic alliances during single subject vs. human partner interactions relative to others.

During the entire briefing the experimenter was standing aside the subject, "helpfully" indicating aloud at the beginning of each series which partner/condition will be encountered. By using this scenario the confederate was unofficially informed when to press the buttons (human condition) and when to relax (computer condition and baseline).

### fMRI setting

After the briefing the experimenter, the confederate and the subject moved to the MR-environment after giving last instructions to the subject and clarifying that the subject understood the winning matrix. In the MR-scanner the same gametask setting as used during the briefing was projected into the MR-compatible video goggles (Resonance Technology). Subjects indicated their decision (cooperate or defect) by pressing one of two buttons with their right hand on a fiberoptic custom-made response box. Prior to each series subjects were informed about the partner/condition they were to play (human, computer or baseline; see Fig. 4). At this point of time the help of the confederate was not needed anymore. With the beginning of the functional imaging recording a randomized script file (the experiment was performed using Presentation^® ^software; Version 0.70, ) was started. The outcomes of each single game were recorded and saved to a computer file. A series of nine games per condition completed one block. Overall, subjects played ten blocks per condition (human partner, computer partner and low level baseline). After scanning subjects were asked to fill out a questionnaire about their impressions of the task and their partners (see Table 1).

### Image Acquisition and Analysis

All scans were performed on a 1.5 T whole body scanner (Phillips Medical Systems, Achieva, Best, Netherlands) using standard gradients and a standard quadrature head coil. Subjects lay in a supine position, while head movement was limited by foam padding within the head coil. In order to ensure optimal visual acuity participants were offered fMRI-compatible glasses that could be fixed to the video glasses. For each subject, we acquired one series of 304 EPI-scans, lasting in total approximately 15 minutes. Stimuli were presented in a blocked design fashion, with ten blocks per condition and a block length of nine single games.

Scans covered the whole brain, including five initial dummy scans parallel to the AC/PC line with the following parameters: number of slices (NS): 31; slice thickness (ST): 4 mm; interslice gap (IG): 4.4 mm; matrix size (MS): 64 × 64; field of view (FOV): 192 mm × 192 mm; repetition time (TR): 2.9 seconds; echo time (TE): 50 ms; flip angle (FA): 90°. For anatomical localization, we acquired high resolution images with a T1-weighted 3D FFE sequence (TR = 25 ms; TE = 4.59 ms; NS = 170 (sagital); ST = 2 mm; IG = 1 mm; FOV = 256 × 256 mm; voxel size = 1 × 1 × 2 mm).

MR images were analyzed using Statistical Parametric Mapping (SPM2, ww.fil.ion.ucl.ac.uk) implemented in MATLAB 6.5 (Mathworks Inc., Sherborn, MA, USA). After discarding the first five volumes, all images were realigned to the first image to correct for head movement. Unwarping was used to correct for the interaction of susceptibility artefacts and head movement. Volumes were then normalized into standard stereotaxic anatomical MNI-space by using the transformation matrix calculated from the first EPI-scan of each subject and the EPI-template. Afterwards, the normalized data with a resliced voxel size of 4 × 4 × 4 mm were smoothed with an 8-mm FWHM isotropic Gaussian kernel to accommodate inter-subject variation in brain anatomy. The time series data were band-pass filtered to remove artefacts due to cardio-respiratory and other cyclical influences.

A general linear model (GLM) comprising three conditions (human partner, computer partner and baseline) was specified for each subject. On the first level, contrasts of main interest were human partner vs. computer partner and vice versa. An SPM2 group analysis was performed by entering these contrast images into random effects analyses using two-sample t-tests to determine between-group analyses. The resulting group contrasts comprised male vs. female (or vice versa) for computer partner > human partner, human partner > computer partner and both conditions vs. baseline. For all group analyses, we applied a voxel-wise threshold of p < 0.001. A Monte Carlo simulation of the brain volume for the current study was conducted to establish an appropriate voxel contiguity threshold [47]. Assuming an individual voxel type I error of p < 0.001, a cluster extent of 6 contiguous resampled voxels was indicated as necessary to correct for multiple voxel comparisons at p < 0.05. The reported voxel coordinates of activation peaks were transformed from MNI space to Talairach & Tournoux atlas space [48] by non-linear transformations .

## Authors' contributions

SK performed the statistical analysis; fMRI analyses and interpretation of data; involved in drafting the manuscript; revising it critically for important intellectual content. IB acquisition of data; made substantial contributions to conception and design. DM acquisition of data; made substantial contributions to conception and design; involved in drafting the manuscript. TL made substantial contributions to conception and design. LK made substantial contributions to conception and design. JW performed the statistical analysis; fMRI analyses. JvO revising it critically for important intellectual content. TK made substantial contributions to conception and design; interpretation of data; involved in drafting the manuscript; revising it critically for important intellectual content.

All authors read and approved the final manuscript.
